# Wnt expression is not correlated with β-catenin dysregulation in Dupuytren's Disease

**DOI:** 10.1186/1477-5751-5-13

**Published:** 2006-08-30

**Authors:** David B O'Gorman, Yan Wu, Shannon Seney, Rebecca D Zhu, Bing Siang Gan

**Affiliations:** 1 Cell and Molecular Biology Laboratory, Hand and Upper Limb Centre, Lawson Health Research Institute, St. Joseph's Health Centre, London, Ontario, Canada; 2 Department of Surgery, University of Western Ontario, London, Ontario, Canada; 3 Department of Physiology and Pharmacology, University of Western Ontario, London, Ontario, Canada; 4 Department of Medical Biophysics, University of Western Ontario, London, Ontario, Canada

## Abstract

**Background:**

Dupuytren's contracture or disease (DD) is a fibro-proliferative disease of the hand that results in finger flexion contractures. Increased cellular β-catenin levels have been identified as characteristic of this disease. As Wnts are the most widely recognized upstream regulators of cellular β-catenin accumulation, we have examined Wnt gene expression in surgical specimens and in DD-derived primary cell cultures grown in two-dimensional monolayer culture or in three-dimensional FPCL collagen lattice cultures.

**Results:**

The Wnt expression profile of patient-matched DD and unaffected control palmar fascia tissue was determined by a variety of complimentary methods; Affymetrix Microarray analysis, specific Wnt and degenerative primer-based Reverse Transcriptase (RT)-PCR, and Real Time PCR. Microarray analysis identified 13 Wnts associated with DD and control tissues. Degenerate Wnt RT-PCR analysis identified Wnts 10b and 11, and to a lesser extent 5a and 9a, as the major Wnt family members expressed in our patient samples. Competitive RT-PCR analysis identified significant differences between the levels of expression of Wnts 9a, 10b and 11 in tissue samples and in primary cell cultures grown as monolayer or in FPCL, where the mRNA levels in tissue > FPCL cultures > monolayer cultures. Real Time PCR data confirmed the down-regulation of Wnt 11 mRNA in DD while Wnt 10b, the most frequently isolated Wnt in DD and control palmar fascia, displayed widely variable expression between the methods of analysis.

**Conclusion:**

These data indicate that changes in Wnt expression *per se *are unlikely to be the cause of the observed dysregulation of β-catenin expression in DD.

## Background

Dupuytren's contracture or disease (DD) is a benign fibro-proliferative disease of the hand that causes permanent finger flexion contractures [1,2]. Despite its long medical history and high prevalence among Caucasians of Northern European ancestry, reportedly as high as 30–40% [3], the underlying genetic etiology of the disease remains unknown [4]. Numerous risk factors have been reported for DD, including alcoholism, trauma, diabetes, smoking, and epilepsy, but their exact role in the disease is not clear [5]. Epidemiological studies show an increased total mortality and cancer mortality rates among men with established DD [6], suggesting the pathophysiology of this disease may overlap with that of certain cancers.

β- catenin, the central component of the 'canonical' Wnt signalling pathway (herein referred to as Wnt/β-catenin) has been implicated in the pathogenesis of DD [7-9], and abnormal β-catenin levels in primary DD cell cultures have been shown to vary with specific cell culture conditions [8,9]. β-Catenin plays both a structural role, as a cadherin-binding protein in cell adhesion junctions [10,11], and a signalling role, as part of the Wnt/β-catenin pathway [12]. Wnts are a large family of lipid modified glycoproteins [13] that regulate various cellular processes important to normal embryonic development [14]. Wnts act as paracrine factors, initiating cell signalling by binding to Frizzled (Fz) receptors. The Wnt/Fz complex can then activate one of three distinct signalling pathways that control either cell fate or differentiation (Wnt/β-catenin)[14], planar cell polarity (PCP)[15], or cell adhesion (Wnt/Ca^+2^/PKC)[16,17]. The co-receptor LRP5/6 (lipoprotein receptor-related proteins 5 or 6) is required for Wnt/β-catenin pathway signalling [18-20]. Once activated, the Wnt/Fz/LRP complex triggers a cascade of signalling events that ultimately lead to the stabilization of a 'cadherin-free' cytoplasmic pool of β-catenin. The cytoplasmic accumulation of β-catenin results in its translocation to the nucleus where it functions as a transcriptional activator for members of the lymphoid enhancer factor/T-cell factor (Lef/Tcf) family of DNA binding proteins [21,22].

The importance of the Wnt/β-catenin signalling is underscored by its targeted disruption in human diseases. For example, several members of the Wnt/β-catenin pathway are mutated in a variety of human malignancies [23-27]. Normally, in the absence of a 'canonical' Wnt signal or an activating mutational event, the cytoplasmic 'free' pool of β-catenin becomes serine/threonine phosphorylated, ubiquitinated (Ub) and degraded in the proteasome, via an axin-based 'destruction' complex. Axin with the aid of APC (adenomatous polyposis coli) binds to β-catenin [28], which facilitates its phosphorylation [29] via a dual kinase mechanism involving CKI (casein kinase-1) and GSK-3β (glycogen synthase kinase-3β) [30-32]. CKI, which is recruited to the destruction complex by the axin binding protein diversin [33], phosphorylates β-catenin at serine 45, an important priming step required by GSK-3β to mediate β-catenin phosphorylation at threonine 41, serine 37 and serine 33. This hyper-phosphorylated form of β-catenin is then recognized by the F-box containing protein slimb/β-TrCP, a component of the E3 ubiquitin (Ub) ligase complex, and β-catenin is targeted for degradation via the 26S proteasome [34-38]. Not surprisingly, the critical serine/threonine residues of β-catenin that are phosphorylated by GSK-3β are mutational 'hot spots' in many cancers. We have previously shown that, unlike the situation in tumors, this region (exon 3) of the β-catenin gene derived from DD samples does not contain such mutations [8]. Given the proposed role of Wnt/β-catenin signalling in DD, in this paper, we set out to examine Wnt expression in DD.

Utilizing multiple approaches, we demonstrate here that multiple Wnts are expressed within patient lesions and control normal palmar fascia (PF) tissue. The pattern of Wnt expression observed in tissue samples is altered by *in vitro *culture method. Comparison of Wnt mRNA levels in DD and control tissues as well as examination of primary cultures of DD cells reveal that the level and type of Wnt expression is highly variable in this fibroproliferative disease with the only consistent finding being down-regulated Wnt 11 mRNA expression in disease tissue. As Wnt-11 signalling is independent of β-catenin and no other Wnt family members display consistent alteration in expression, this data suggests other, as yet unidentified, factors are dysregulating β-catenin processing in DD.

## Results

### Affymetrix microarray analysis

**Figure 1:**
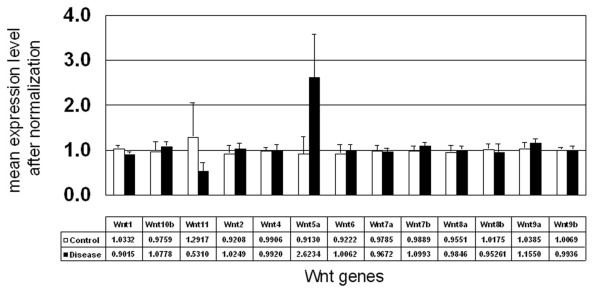
**Affymetrix microarray of Wnt expression**. 3 DD and 3 control samples were analyzed using the Human Genome U133 Plus 2.0 Array. The data generated represents the expression analysis from all samples. The data was sorted and normalized using Genespring software. The control value (mean of three samples relative to normalization of control #1 and corresponding disease value (mean of three samples) is shown beneath each Wnt designation on the X axis. The graph displays the mean values of each group ± standard deviation.

The primary goal of this project was to determine the expression status of all Wnts expressed in DD and control palmar fascia (PF). To achieve this, surgically resected DD and control patient samples were examined using Affymetrix Microarray as described in the methods. As shown in Figure 1, 13 of the 19 Wnts (for a review of Wnt factors: ) were identified as being expressed in DD. Data indicated that the majority of Wnts detected in both DD and control were expressed at very similar levels with only Wnts 5a and 11 displaying any variance in expression between disease and control tissues.

### Wnt expression profiling *in vivo *and *in vitro *using Wnt specific primers

**Figure 2:**
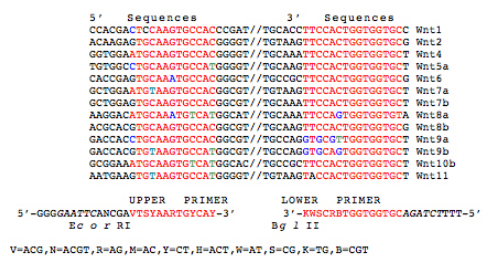
**Degenerate primer design**. Degenerative Wnt primers (designated Upper and Lower Primers) were designed using the ClustalW program with the aid of the BioEdit sequence alignment editor. Comparison of the published nucleic acid sequences for the 13 human Wnts detected by Affymetrix Microarray revealed two highly conserved regions that were used to design degenerate oligonucleotide primers for PCR amplification as shown.

**Figure 3:**
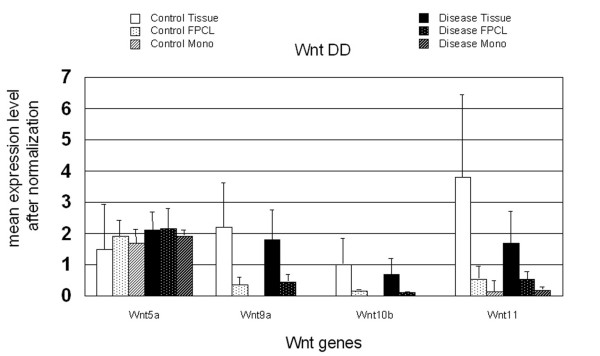
**Comparison of Wnt expression in RNA derived from tissue, FPCL and monolayer culture**. Comparisons of the *in vivo *and *in vitro *expression levels of Wnts 5a, 9a, 10b, and 11 were performed by RT-PCR using the Wnt-specific oligonucleotide primers from RNA derived from DD and PF control samples. As shown, a distinct expression hierarchy (Tissue > FPCL > monolayer) was shared by Wnts 9a, 10b and 11 with Wnts 9a and 10b being undetected in monolayer culture. Wnt5a expression levels were unaffected by culture conditions. In all cases Wnt expression was normalized to GAPDH expression and represent the mean value ± standard deviation.

To confirm the Microarray data and to assess the relative contribution of each of these 13 Wnts in DD and control PF, a Wnt expression profiling study was initiated utilizing Wnt degenerate primer analysis. Prior to commencing these experiments, however, it was necessary to determine the optimal samples for analysis. We have previously reported that β-catenin levels are abnormal in DD tissue but not in primary cell isolates grown in two-dimensional monolayer culture, whereas three-dimensional culture of the same primary cell lines in FPCL can recapitulate abnormal β-catenin expression [8,9]. The initial experiments involved the isolation of total RNA from surgically resected patient samples and primary cell cultures grown either in two-dimensional monolayer culture or in three-dimensional FPCL culture as described in the methods. Total RNA samples were then reverse transcribed and the cDNA templates were amplified by PCR using Wnt degenerate oligonucleotide primers (Fig. 2). Clones of the PCR amplified products were isolated, and the cloned inserts from purified plasmid DNA digested with a series of diagnostic restriction enzymes to identify the corresponding Wnt subtype as described in the methods. Clones were categorized based on the Wnt-specific DNA fragment sizes, and several representative clones from each group sequenced. The clones isolated from control and disease PF tissue displayed a similar Wnt expression pattern, with Wnt 5a, 9a, 10b, and 11 accounting for almost all of the identifiable disease and control clones detected by this approach (as shown in Table 1). Two clones representing Wnts 8b and 9b were also isolated. Comparisons of the *in vivo *and *in vitro *expression levels of the major group of Wnts (5a, 9a, 10b, and 11) were performed by RT-PCR using the Wnt-specific oligonucleotide primers as described in the methods. As shown in Figure 3, three of the four Wnts (5a, 9a and 11) were readily detectable within FPCL cultures, while only two Wnts (5a and 11) were detected in confluent monolayer cultures. Wnt5a expression levels were relatively high for both monolayer and FPCL cultures and quite similar to the levels seen *in vivo*. However, comparisons between the *in vivo *and *in vitro *expression levels of Wnt 9a, 10b and 11 showed significant differences. Specifically, a distinct expression hierarchy (*in vivo *> FPCL > confluent monolayer) was shared by Wnts 9a, 10b and 11. Wnt10b expression was very low or undetectable in the majority of the FPCL cultures and largely undetectable in the confluent monolayer cultures.

**Table 1:** Wnt expression profiling of surgical fascia tissue specimens.

*Patient*	*Fascia Type*	*Wnt 5a*	*Wnt 9a*	*Wnt 10b*	*Wnt 11*
D2	Control	5/37	9/37	3/37	20/37
Pooled	Control	4/17	0/17	5/17	8/17
W	Control	1/3	2/3	0/3	0/3
D	Disease	0/28	1/28	27/28	0/28
D2	Disease	1/4	0/4	2/4	1/4
E	Disease	0/16	0/16	16/16	0/16
M	Disease	0/9	0/9	9/9	0/9
H2	Disease	0/16	0/16	16/16	0/16
L	Disease	0/2	1/2	1/2	0/2
R	Disease	2/9	0/9	7/9	0/9
T	Disease	5/22	1/22	10/22	6/22
W	Disease	11/19	0/19	3/19	8/19

In total, 9 disease, 7 control or normal fascia specimens (2 individual + 5 that were 'pooled' together) and were subjected to RT-PCR using degenerate Wnt primers. Four different Wnt species were identified; Wnt5a, 9a, 10b and 11 which were isolated from 18%, 19%, 14% and 49% of the control clones (n = 57), and 15%, 2%, 73% and 12% of the disease clones (n = 125), respectively (total 182)

Based on this data, we concluded that these *in vitro *culture conditions do not recapitulate *in vivo *Wnt expression and that only RNA isolated directly from surgical specimens would be suitable to obtain an accurate representation of Wnt expression in DD.

### Wnt expression profiling in DD and control palmar fascia by reverse transcription and degenerate primer PCR analysis

In light of the results from the *in vitro *and *in vivo *studies described above, a comprehensive Wnt expression profiling study was performed on RNA isolated directly from surgical specimens utilizing reverse transcription and Wnt degenerate primer PCR analysis. PCR products (~400 bp) were subcloned into the pCR^® ^4-TOPO^® ^sequencing vector and clones were isolated as described in the methods. The DNA restriction analysis was performed and a representative subgroup of clones were sequenced to confirm Wnt identity. In total, 182 clones were isolated and identified by restriction enzyme analysis to yield a representative overview of Wnt expression in DD and control palmar fascia. As shown in Table 1, Wnts 5a, 9a, 10b, and 11 accounted for all clonal isolates. Specifically, Wnts 5a, 9a, 10b, and 11 represented 18%, 19%, 14% and 49% of the control clones (n = 57), and 15%, 2%, 73% and 12% of the disease clones (n = 125), respectively (total 182).

### Real Time PCR of Wnt 10b and Wnt 11 expression in DD and control palmar fascia

**Figure 4:**
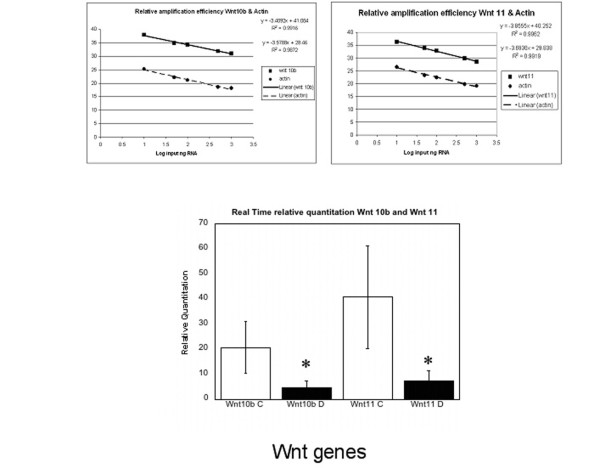
**Real time relative quantitation of Wnt 10b and Wnt 11 expression in DD vs. control**. Real time relative quantitation of PCR amplicons was performed on an ABI Prism 7700 using the comparative Ct method. UPPER PANELS: Determination of relative amplification efficiencies for Wnt10b vs. β-actin and Wnt11 vs. β-actin. As shown, target and endogenous control gene products amplified with similar efficiency. The Δ C_T _value (C_T__target_/C_T__endogenous control_) was determined to be 0.1696 and 0.1619 for Wnt 10b/β-actin and Wnt 11/β-actin respectively. LOWER PANEL: Real time relative PCR quantitation for Wnt10b and Wnt11 mRNAs performed on a separate subset of 7 DD and 6 PF control samples derived from surgical resection. Triplicate reactions of each dilution were performed (50 μl samples) in a 96-well plate format using SDS instrumentation (Applied Biosystems) for 45 cycles. Target and endogenous control reaction were run in separate wells in triplicate at each concentration. Relative Quantitation of Wnt 10b and Wnt 11 mRNA levels in normal palmar fascia (mean Wnt 10b = 20.74 ± 8.81, Wnt 11 = 40.89 ± 12.86) were significantly (*) different to DD (Wnt 10b = 4.83 ± 2.21; P = 0.038, Wnt 11 = 7.56 ± 4.50; P = 0.014, Mann Whitney Test). Data is shown as Mean ± SEM.

Wnts 10b and 11 represented the majority of clonal isolates from DD and control tissues and their representation within each group appeared to correlate with the presence of disease. To better compare the expression levels of these Wnts, we performed Real Time PCR of Wnt 10b and Wnt 11 mRNA in surgical samples of DD and normal PF control using the relative quantitation method. As shown in Figure 4, both Wnt 10b and Wnt 11 mRNA levels were significantly decreased in total RNA derived from DD cord (Mann-Whitney Test, Wnt 10b P = 0.038, Wnt 11 P = 0.014). The expression levels of Wnt10b did not reflect the representation in the clones derived from the degenerate primer analysis or the Affymetrix Microarray analysis. Wnt 11 mRNA expression, by comparison, was lower in DD tissue than control palmar fascia by all of the techniques employed. With the exception of Wnt 11, direct comparison of the Wnt expression analysis reported here indicated that the level and type of Wnt expression is not altered between DD and control PF.

## Discussion

Wnt expression is altered in a variety of cancers including prostate and colon carcinoma [39,40], where β-catenin accumulation and associated gene transcription are believed to contribute to the tumor growth. As we and others have previously shown that abnormal cytoplasmic accumulation of β-catenin is present in DD, we have hypothesized that β-catenin is a central molecular mediator of its pathophysiology [7-9]. As Wnt factors are the most upstream regulating factors of β-catenin levels, we have undertaken a comprehensive analysis of Wnt expression in this disease.

Of the 19 Wnts identified to-date, 13 were detected by Affymetrix Microarray analysis in both DD and PF control tissue. Normalized expression analysis indicated that the majority of Wnts were expressed at equivalent levels in both disease and control samples with only Wnt 11 and Wnt 5a displaying any suggestion of altered expression. A large standard deviation was noted in the control samples for Wnt 11 (mean 1.29, SD 0.72) and the disease samples for Wnt5a (mean 2.62, SD 1.05). The Wnt expression of the control sample derived from a patient undergoing carpal tunnel release was not significantly different from the Wnt expression of the control samples derived from palmar fascia adjacent to disease cord in all cases including Wnt 11, where it was the sample closest to the mean (data not shown). As such, none of the variability in Wnt expression was readily attributable to genetic background.

The primary advantage of the Microarray approach is the ability to sensitively screen the expression levels of a large number of mRNA transcripts in a process with low between-assay variability. This tool is designed to compare samples, in this case DD and control PF, and the readout is designed so that control readings are normalized and disease values are reported relative to that normalized value. Thus, in this study the Microarray analysis yielded an accurate and comprehensive overview of Wnt expression in the samples of DD and control PF without indicating the relative abundance of each Wnt subtype within each category. While this data indicated that both Wnt 5a and Wnt 11 expression were potentially altered in DD, it was unclear what percentage of the total Wnt signalling potential of the cells was represented by these two transcripts.

To more rigorously determine the relative contributions of the 13 Wnt transcripts detected, we therefore continued our analysis by determining the Wnt expression profile utilizing degenerate primers. Progressive multiple sequence alignment of the Wnt gene family using ClustalW [41] revealed several highly conserved regions that could be used to design degenerate oligonucleotide primers as previously described [42], that would be able to amplify all 13 Wnts identified in the microarray. This approach has the advantage of being amenable to assessing large numbers of samples with the options of restriction enzyme analysis and/or sequence level identification of the clonal isolates. Further, as each cDNA is generated from the total RNA pool, the amplified products would be predicted to be isolated at a frequency proportional to the initial abundance of the mRNA transcripts.

Initial Wnt expression profiling experiments were directed at determining the appropriate sample for analysis. This was essential, as the central hypothesis of this study was that alterations in Wnt signalling could be the primary cause of the altered cellular accumulation of β-catenin evident by immunohistochemistry of DD tissue. We have previously reported that this accumulation of β-catenin in DD was not detected by western blotting of primary cell isolates grown in two-dimensional monolayer culture [8]. Three-dimensional cultures of the same primary cell lines in Fibroblast Populated Collagen Lattices (FPCL), however, were able to qualitatively reflect *in vivo *β-catenin expression due, at least in part, to the addition of appropriate isometric tension [9]. As this study was inherently quantitative, it was of interest to determine the effect of isometric tension and three-dimensional FPCL culture on the level of Wnt expression and to determine how Wnt expression level in FPCL compared to Wnt expression *in vivo*.

The analysis of clones by restriction analysis and sequencing revealed that Wnts 5a, 9a, 10b, and 11 accounted for the vast majority of the disease and control-derived clones that could be identified by this approach. Comparisons of the *in vivo *and *in vitro *expression levels of these Wnts (5a, 9a, 10b, and 11) were performed by RT-PCR using the Wnt-specific oligonucleotide primers. Three of the four Wnts (5a, 9a and 11) were readily detectable within FPCL cultures, while only two Wnts (5a and 11) were detected in confluent monolayer cultures. Wnt 5a expression levels were relatively high for both monolayer and FPCL cultures and quite similar to the levels seen *in vivo*. However, comparisons between the *in vivo *and *in vitro *expression levels of Wnt 9a, 10b and 11 showed significant differences. Specifically, a distinct expression hierarchy (*in vivo *> FPCL > confluent monolayer) was shared by Wnts 9a, 10b and 11. Wnt10b expression was very low or undetectable in the majority of the FPCL cultures and largely undetectable in the confluent monolayer cultures.

Based on these data, we concluded that these *in vitro *culture conditions do not recapitulate *in vivo *Wnt expression and that RNA isolated directly from surgical specimens is required to obtain an accurate representation of Wnt expression in DD. A comprehensive Wnt expression profile of 182 clones was generated utilizing reverse transcription and Wnt degenerate primer PCR analysis. As shown in Table 1, Wnt 5a was evident in 15% of DD- derived clones and 18% of control derived clones, indicating that there was no difference in expression level between these groups. In contrast, the relative abundance of clones containing Wnts 10b and 11, and to a lesser extent 9b, sequences varied considerably between DD and controls. Wnt 11 was evident in 49% of control tissue – derived clones but only 12% of those derived from disease tissue, whereas Wnt 10b displayed the opposite trend being present in only 14% of control-derived clones compared to 73% of DD-derived clones. Wnt10b is recognized to primarily signal via the "canonical" pathway leading to downregulation of GSK-3β activity and cytoplasmic accumulation of β-catenin [43]. Wnt 11, while less well characterized, has been shown to be associated with the "non-canonical" cell adhesion (Wnt/Ca^+2^/PKC) pathway [44]. As this data could imply a shift to increased Wnt 10b expression and decreased Wnt 11 expression in DD, consistent with an increase in signalling through the canonical pathway, it was essential to independently confirm these data.

To achieve this, Wnt 10b and Wnt 11 expression were quantitated by Real Time PCR on an additional subset of 7 DD and 6 PF control samples derived directly from surgical resection. As shown in Figure 4, these results indicate that Wnt 10b expression was significantly decreased between disease and control samples. The lack of correlation between Wnt 10b expression in the Affymetrix Microarray analysis, which indicated no change between DD and control samples, the degenerate primer analysis, which indicated an increased abundance of cDNAs derived from Wnt 10b mRNA, and the decreased Wnt 10b mRNA levels revealed by Real Time PCR data, was unexpected. These contradictory findings dictate that an exclusion of changes in Wnt 10b mRNA expression in the pathophysiology of DD cannot be made at this time. Wnt 11 mRNA expression was also variable, however in this case a consistent decrease in expression between disease and control samples was evident in all of the analytical techniques employed. Wnt 11 is reported to signal through a non-canonical pathway that does not affect β-catenin accumulation [44,45]. The absence of consistent changes in the mRNA expression of any other Wnts identified in DD that could lead to a shift to canonical Wnt signalling indicates that, accounting for variability between individual samples, there is no evidence of a consistent alteration in Wnt mRNA expression in DD that would alter β-catenin accumulation.

While Wnt expression is unchanged, it should be noted that these data do not rule out alterations in Wnt signalling in the pathogenesis of this disease. Wnt signalling efficiency has been shown to be altered in a variety of tumors secondary to changes in the expression of secreted Wnt antagonists, such as the Dickkopf family, Wnt inhibitory factor-1 and secreted Frizzled-related protein (sFRP) family [46-48]. Our Affymetrix Microarray data indicated that there was a consistent reduction in sFRP-1 expression in the 3 DD samples screened relative to PF control levels (data not shown). It is possible, therefore, that alterations in total (rather than individual) Wnt signalling activity may be affected by down-regulation of sFRP-1 in DD. Importantly, post-translational modification of Wnt/β-catenin signalling pathway components, such as altered phosphorylation, acetylation, methylation, ubiquitination, sumoylation, glycosylation or lipidation, could contribute to an altered responses of DD cells to upstream signalling molecules. In addition, while we have shown that exon 3 mutations of the β-catenin gene are not evident in DD [8], we have not ruled out the possibility that changes to other regions of this or other genes encoding integral components of this pathway such as APC may be altering the sensitivity of DD cells to Wnt signalling. We are presently assessing the activity of GSK-3β in DD to determine if upstream signalling molecules are regulating kinase activity and cytoplasmic stability of β-catenin in this fibroproliferative disorder.

## Conclusion

Wnt gene expression was shown to be affected by *in vitro *culture, both in routine monolayer culture and in three dimensional culture in FPCL, when assessed by RT-PCR. Affymetrix Microarray analysis, degenerate primer analysis and Real Time relative PCR quantitation were performed and, with the exception of Wnt 10b where expression was highly variable between analyses, the data indicate that overall Wnt expression is unchanged between samples derived from surgical specimens of DD and those derived from normal PF. As such, alterations in the expression levels of individual Wnt subtypes are unlikely to be contributing to the observed dysregulation of β-catenin expression in DD.

## Methods

### Clinical specimen collection

DD patient specimens (normal and disease PF) were collected in compliance with the University's Human Research Ethics Committee. Disease cords and nodules (disease) and uninvolved normal fascia (control) were collected from patients undergoing surgical resection of DD lesions as following: All samples used in the current study were from primary resections. In the operating room, the superficial surface of the affected palmar fascia including a surrounding area of normal appearing fascia was widely exposed. The diseased part was subsequently resected with a cuff of normal appearing palmar fascia. On the side table, the diseased cord was then removed from the resected specimen and immediately sent to the lab. In addition, an area of uninvolved normal appearing fascia (control) well-away from the resected specimen was harvested. To incorporate a "true normal", palmar fascia from patients who do not have any signs of Dupuytren's disease was harvested. Thus, one sample from a patient undergoing carpal tunnel release was also utilized in the Affymetrix Microarray study. The majority of clinical specimens were processed immediately for total RNA isolation as described below, unless otherwise indicated, in which case samples were stored at -80°C prior to processing.

### Affymetrix microarray analysis

Surgical specimens were transferred to dry ice immediately after clinical dissection. Approximately 100 mg tissue samples were minced into small pieces on dry ice and then snap-frozen in Liquid Nitrogen. Tissue samples were ground in a mortar and pestle and 1 ml TRIzol reagent (Invitrogen Canada Inc., Burlington, Ontario) was added until all the powder dissolved. Samples were transferred to 1.5 ml microcentrifuge tubes and total RNA was isolated using TRIzol procedure. Total RNA was purified and stabilized using RNeasy Minikit (Qiagen Inc., Mississauga, Ontario). Aliquots (3 μl) were screened using an Agilent 2100 Bioanalyzer (Agilent Technologies, Mississauga, Ontario) and high quality RNA samples were submitted to the London Regional Genomic Center  for Microarray analysis on a Human Genome U133 Plus 2.0 Array (Affymetrix, Santa Clara, CA). The data generated represents the expression analysis from all samples (3 DD compared to 3 control consisting of 2 PF and 1 carpal tunnel release). The data was analyzed using Genespring software (Agilent Technologies, Mississauga, Ontario).

### Primary cell monolayer culture

Primary cell cultures were established as previously described [9,49]. Initially, primary cell cultures were grown in starter media containing α-MEM (Gibco, Invitrogen Corporation), 20% fetal bovine serum (FBS, Clontech Laboratories, Palo Alto, CA), penicillin G + streptomycin sulfate, and fungizone (Gibco, Invitrogen Corporation). Established primary cell cultures were maintained in α-MEM + 10% FBS + antibiotics + fungizone at 37°C in a humidified chamber with 5% CO_2_.

### Fibroblast populated collagen lattice (FPCL) cultures

Collagen lattices were prepared as a modified version of the method described previously [8]. Briefly, phosphate buffered solution (PBS) suspensions of primary cell cultures (passages 2 – 6) were mixed with a neutralized solution of Vitrogen100 (Collagen Corp, Santa Clara, CA, USA) collagen type I matrix (8 parts Vitrogen100, 2.9 mg/ml + 1 part 10× α-MEM + 1 part HEPES buffer, pH 9.0). The cell-collagen concentrations were adjusted with PBS to attain a final collagen concentration of 2.0 mg/ml and a final cell concentration of 10^5 ^cells/ml of matrix. The cell-collagen mixture was then dispensed into 24 well culture dishes (0.5 ml/well) that were pre-treated with a PBS solution containing 2% (w/v) bovine serum albumin (BSA). Following FPCL polymerization, 0.5 ml of growth medium (α-MEM, 10% FBS) was added on top of each lattice. After 4 days of culture the attached FPCLs were harvested for RNA extraction using the RNeasy^® ^columns according to the manufacturer's instructions (Qiagen, Mississauga, ON, Canada).

### Degenerate Wnt primer design

Progressive multiple sequence alignment of the Wnt gene family was carried out using the ClustalW program with the aid of the BioEdit sequence alignment editor [41]. Comparison of the published nucleic acid sequences for the 13 human Wnts detected by Affymetrix Microarray revealed several highly conserved regions that were used to design degenerate oligonucleotide primers for PCR amplification as previously described [42].

### RNA extraction and PCR amplification of Wnt genes using degenerate oligonucleotide primers

RNA extraction from tissues was carried out as previously described. First-strand cDNA was reverse transcribed (RT) from 1 mg of total RNA using random hexamer priming and SuperScript™ II reverse transcriptase in a final volume of 50 ml, as recommended by the manufacturer (Invitrogen, Carlsbad, CA, USA). Following first strand synthesis, cDNA was amplified using the following degenerate oligonucleotide primers:

(UP) upper primer 5'-GGGGAATTCANCGAVTSYAARTGYCAY-3'

(LP) lower primer 5' – AAAAGATCTGCACCACCAVYGSWM-3'

where V = ACG, N = ACGT, R = AG, M = AC, Y = CT, H = ACT, W = AT, S = CG, K = TG and B=CGT

Standard PCR amplification was performed in a PX2 Thermal Cycler (Thermo Electron Corporation) using 4 μl of RT cDNA, 1 mM MgCl_2_, 200 μM of dNTPs (Invitrogen, Carlsbad, CA, USA), 1 μM of primers, and 1 unit of Platinum^® ^Taq DNA Polymerase (Invitrogen, Carlsbad, CA,.USA) in Taq PCR buffer (10 mM Tris pH8.3, 50 mM KCl). PCR cycling parameters were 94°C for 5 min, followed by 40 cycles of 94°C for 1 min., 59°C for 1.5 min., 72°C for 2 min., followed by a final extension at 72°C of 10 min.

### Cloning of PCR products and screening of transformants

PCR products (~400 bp) were directly subcloned into the pCR^® ^4-TOPO^® ^sequencing vector using a TOPO TA cloning kit, as recommended by the manufacturer (Invitrogen). Ligation products were then used to transform TOP10 chemically competent *E. coli *cells, and transformants selected for using standard ampicillin (100 μg/ml) supplemented LB agar plates. Plasmid DNA was purified from selected colonies using Qiagen mini-prep columns (Qiagen, Mississauga, ON, Canada). Plasmid DNA was quantified by spectrophotometry (OD_260_), digested with *Eco*RI (pCR4-TOPO subcloned amplicons are flanked by *Eco*RI sites), and subjected to 2% agarose gel electrophoresis. DNA bands were gel purified (Qiagen gel extraction kit) and then digested with a series of diagnostic restriction endonuclease (RE), including *Eco *RI, *Eco *RII and *Sac *II. DNA fragments were then size-separated using native (non-denaturing) acrylamide (12%) gel electrophoresis. Gels were stained with ethidium bromide, placed on a UV transilluminator (FisherBiotech) and photographed using Polaroid film (type 667) and a Polaroid DS-34 camera fitted with a UV filter (Polaroid, UK). The DNA fragmentation patterns were analyzed (i.e. RE grouping), and several clones from each RE group were sequenced to confirm their Wnt identity.

### RT-PCR using Wnt-specific primers

Briefly, 200 ng of total RNA from tissue or cells were reverse transcribed using random hexamers and SuperScript™ II RT as described above (Invitrogen, Carlsbad, CA, USA). Following first strand synthesis, Wnt specific PCR amplifications were carried out using 1 μl of cDNA, 1.5 mM MgCl_2_, 200 μM of dNTP mix (Invitrogen, Carlsbad, CA, USA), 0.8 μM of primers (see below), and 0.75 units of Platinum^® ^Taq DNA Polymerase (Invitrogen, Carlsbad, CA., USA) in Taq PCR buffer (10 mM Tris pH8.3, 50 mM KCl), in a final reaction volume of 25 μl. Sequences of the Wnt-specific primer were as following:

Wnt5a upper primer (Wnt5a-UP): 5' – aagaagtgcacggagatcgt – 3'

Wnt5a lower primer (Wnt5a-LP): 5' – tggaacctacccatcccata – 3'

Wnt9a upper primer (Wnt9a-UP): 5' – gcaagcatctgaagcacaag – 3'

Wnt9a lower primer (Wnt9a-LP): 5' – tgctctcgcagttcttctca – 3'

Wnt10b upper primer (Wnt10b-UP): 5' – ctggtgctgctatgtgctgt – 3'

Wnt10b lower primer (Wnt10b-LP): 5' – cccagccaaaaggagtatga – 3'

Wnt11 upper primer (Wnt11-UP): 5' – tgacctcaagacccgatacc – 3'

Wnt11 lower primer (Wnt11-LP): 5' – tgagggtccttgagcagagt – 3'

GAPDH upper primer: 5' – gtcagtggtggacctgacct – 3'

GAPDH lower primer: 5' – aggggtctacatggcaactg – 3'

PCR cycling parameters were optimized for Wnt5a, Wnt9a, Wnt10b, Wnt11 and GAPDH to ensure log linear amplification of these products. For Wnt5a, Wnt9a, Wnt10b, Wnt11 were 94°C for 3 min, followed by 32 cycles of 94°C for 30 sec., 62°C for 45 sec., 72°C for 45 sec., followed by a final extension at 72°C of 7 min. PCR conditions for GAPDH were the same as above except 25 cycles were used. PCR samples were then subjected to 2% agarose gel electrophoresis, with the resulting amplicons being visualized by ethidium bromide staining. Digital images of the gels were captured and analyzed using a gel documentation workstation (Alpha Innotech Corp., San Leandro, CA, USA).

### Real Time PCR

Real time PCR was performed on an ABI Prism 7700 (Applied Biosystems, Foster City, CA. USA) using the relative quantitation, or "comparative Ct" method. In brief, TRIzol reagent (Invitrogen) and RNeasy^® ^Mini Kits (Qiagen, Mississauga, ON) were used to isolate total RNA from surgically resected normal and diseased tissue from patients with Dupuytren's Contracture. RNA quality was determined on an Agilent 2100 Bioanalyzer and only samples with minimal degradation were used for analysis. 10 μg of total RNA was reverse transcribed into cDNA first strand using the High-Capacity cDNA Archive Kit (Applied Biosystems) in accordance with the manufacturer's instructions. For validity, the comparative Ct method requires that the amplification efficiency of the target and endogenous control transcripts be equivalent. To assess this, dilutions of cDNA first strand corresponding to 1000 ng, 500 ng, 100 ng, 50 ng, 10 ng and 0 ng were introduced into the PCR reactions. Coupled with the target genes Wnt 10b and Wnt 11, endogenous control gene products were amplified by gene specific probe sets containing TaqMan^® ^MGB probes labelled with 6-FAM™ (Applied Biosystems, Wnt 10b Assay Id Hs00559664_m1; Wnt 11 Assay ID Hs00182986_m1). Triplicate reactions of each dilution were performed (50 μl samples) in a 96-well plate format using SDS instrumentation (Applied Biosystems) for 45 cycles. Target and endogenous control reaction were run in separate wells in triplicate at each concentration. To determine if the target and endogenous control gene products amplified with the same efficiency, the Δ C_T _value (C_T __target_/C_T __endogenous control_) was plotted against the log input cDNA to create a semi-log regression line. A "pass" value of <0.2 for the slope of Δ C_T _vs. log input was used in this study. β-actin amplification passed validation with Δ C_T _vs. log input values of 0.1696 and 0.1619 for Wnt 10b and Wnt 11 respectively, and this control was subsequently used for all real time relative quantitation in this study.

## Competing interests

The author(s) declare that they have no competing interests.

## Authors' contributions

YW, SS and RDZ carried out (RT-) PCR, DNA microarray and cell culture, as well as first interpretation of the data. DBO and BSG are responsible for study conception and design, coordinated the entire project, performed final interpretation of the data and completed the manuscript. All authors read and approved the final manuscript.
